# Periapical Bone Loss as a Signal of Risk for Prosthetic Screw Loosening in Full-Arch Implant-Supported Fixed Complete Dentures: A Multicenter Retrospective Pilot Study

**DOI:** 10.7759/cureus.110923

**Published:** 2026-06-15

**Authors:** Pawaris Ngamdumrongkiat, Ramon Urapepon

**Affiliations:** 1 General Practice, Srinakharinwirot University, ฺBangkok, THA; 2 General Practice, Srinakharinwirot University, Bangkok, THA

**Keywords:** all-on-4, all-on-6, dental implants, full-arch fixed complete dentures, implant prosthodontics, periapical bone loss, prosthetic screw loosening, retrospective pilot study

## Abstract

Purpose: Prosthetic screw loosening is among the most prevalent mechanical complications in full-arch implant-supported fixed complete dentures (FCDs). This multicenter retrospective pilot study aimed to detect a preliminary signal of association between periapical bone loss (PBL) characteristics and prosthetic screw loosening in All-on-4 and All-on-6 FCDs and to generate effect-size estimates for planning future confirmatory studies.

Materials and methods: Forty-three arches from 32 patients at three private dental clinics in Bangkok, Thailand (2017-2025) were retrospectively analyzed. PBL was assessed on panoramic radiographs; intraexaminer reliability was evaluated using the intraclass correlation coefficient (ICC). Eleven predictor variables were examined by univariate logistic regression. Firth's penalized logistic regression was performed as the primary multivariable analysis, with Kaplan-Meier survival analysis as a supplementary time-to-event analysis.

Results: Screw loosening occurred in 10 of 43 arches (23.3%). The intraexaminer ICC was 0.990. The number of PBL-affected implants showed a significant signal of association with screw loosening in univariate analysis (OR=2.539, 95% CI:1.200-5.371, P=.015). Kaplan-Meier analysis demonstrated significantly shorter time to first screw loosening in arches with ≥3 PBL-affected implants versus 1-2 (19.5 vs 30.0 months; log-rank P=.002).

Conclusions: Among PBL-affected arches, the number of PBL-affected implants per arch showed a signal of association with prosthetic screw loosening across multiple analytical approaches. These exploratory, hypothesis-generating findings warrant confirmation in adequately powered prospective studies.

## Introduction

Implant-supported fixed complete dentures (FCDs), configured as All-on-4 or All-on-6 reconstructions, have become a standard of care for the rehabilitation of complete edentulism, providing predictable long-term survival and substantial improvements in masticatory function and oral health-related quality of life relative to removable alternatives [1,2]. Nevertheless, prosthetic screw loosening remains the most frequently reported mechanical complication, with incidence rates of approximately 2.8% to 33% across published cohorts, varying with follow-up duration, prosthetic design, and occlusal loading conditions [3-8]. When left unaddressed, screw loosening compromises prosthesis stability, necessitates unplanned clinical intervention, and may escalate to more severe sequelae including screw fracture and framework failure [9].

Peri-implant bone support is a critical determinant of the long-term biomechanical integrity of the implant-prosthesis complex. To date, most investigations of bone-related risk factors for prosthetic complications have concentrated on marginal bone loss and peri-implantitis [10-13]. Comparatively little attention has been directed toward periapical bone loss (PBL), a clinically and radiographically distinct entity defined as localized resorption of alveolar bone surrounding the apical portion of an osseointegrated implant, presenting as a well-defined radiolucent area at or near the implant apex while coronal bone-to-implant contact remains intact [14-16]. This condition has been described under various terms, including retrograde peri-implantitis, implant periapical lesion, and apical peri-implantitis, with reported prevalence ranging from 0.26% to 3.7% in general populations to as high as 23.8% in high-risk cohorts such as implants placed at sites with preexisting periapical pathology [14-18]. This wide range reflects differences in the population sampled (general versus high-risk cohorts such as sites with preexisting periapical pathology), the imaging modality and diagnostic threshold used (cone-beam computed tomography (CBCT) detecting lesions missed on panoramic radiography), and inconsistent case definitions across terminology, rather than true biological variation alone. In this manuscript, PBL is used as the umbrella radiographic descriptor for this entity; the terms retrograde peri-implantitis, implant periapical lesion, and apical peri-implantitis are etiologic-clinical labels applied to the same radiographic finding, and PBL is used consistently throughout to denote the radiographic finding independent of presumed etiology.

Prior work on PBL/retrograde peri-implantitis has focused chiefly on its prevalence, etiology, and treatment [14-19], whereas bone-related risk factors for prosthetic complications have been examined almost exclusively in relation to marginal bone loss and peri-implantitis [10-13]. No study has investigated PBL specifically in relation to mechanical complications of full-arch FCDs, providing the rationale for the present pilot.

The etiology of PBL is multifactorial. Bacterial contamination from a preexisting periapical infection at the recipient site or from an endodontically compromised adjacent tooth represents the most common infectious cause [18,19]. Aseptic etiologies include thermal bone necrosis during osteotomy preparation, bone compression from excessive insertion torque, implant surface contamination, and intraoperative vascular compromise [17,19]. Regardless of the initiating mechanism, the shared pathologic endpoint is progressive periapical bone resorption, which reduces the effective bone-to-implant contact length and diminishes the anchorage capacity of the affected implant [14-17].

From a biomechanical perspective, the stability of the prosthetic screw joint depends on the preload-the axial clamping tension generated when the screw is tightened to a specified torque [20,21]. Maintaining adequate preload requires a rigid implant-bone interface capable of resisting functional loading without excessive micromotion [21-23]. When periapical anchorage is compromised by PBL, the affected implant becomes more susceptible to deflection under occlusal forces, generating increased bending moments and nonaxial stress concentrations at the implant-abutment interface that may progressively erode screw preload [22,23]. In full-arch fixed reconstructions, this biomechanical consequence may be amplified: cumulative PBL affecting multiple implants within a single prosthetic unit can disproportionately redistribute occlusal forces across the remaining screw joints, compounding nonaxial stress cycles and potentially accelerating fatigue-related preload loss [22,23].

Importantly, the relationship between PBL and prosthetic screw loosening may not be unidirectional. Experimental evidence in a canine model has demonstrated that the presence of a microgap at the implant-abutment interface elicits persistent acute inflammatory infiltration, predominantly neutrophilic, in the adjacent connective tissue, and that two-piece implants with such microgaps exhibit significantly greater crestal bone loss than one-piece designs lacking an interface [24]. A comprehensive review of the implant-abutment interface literature further confirmed that microgap and micromotion at the junction directly and indirectly induce microleakage and mechanical damage, progressively enlarging the gap and culminating in marginal bone resorption [25]. By extension, prosthetic screw loosening, which enlarges the functional microgap and introduces restoration micro-movement, may facilitate microbial colonization of the implant-abutment interface, triggering peri-implant inflammation that contributes to further bone loss [9,20,24-26]. No previous study has explicitly examined this potential bidirectional or self-reinforcing cycle between bone loss and prosthetic screw loosening in any implant prosthesis configuration; to date, the concept remains a theoretically plausible but untested hypothesis.

A comprehensive search of PubMed, Scopus, and Google Scholar databases (conducted in January 2025 using the terms "periapical bone loss," "retrograde peri-implantitis," "implant periapical lesion," combined with "screw loosening" and "fixed complete denture") identified no published study that has specifically investigated the relationship between PBL and screw loosening in full-arch FCDs. The purpose of this exploratory pilot study was therefore to examine the association between PBL, characterized by the number and anatomical position of PBL-affected implants per arch, and prosthetic screw loosening in All-on-4 and All-on-6 FCDs, and to generate preliminary effect-size estimates to inform sample-size calculations for future confirmatory investigations. The null hypothesis tested was that neither the number nor the anatomical position of implants exhibiting PBL would demonstrate a statistically significant association with prosthetic screw loosening. Because the design is case-only, the present analysis evaluates variation within PBL-positive arches rather than comparing PBL-positive with PBL-free patients.

## Materials and methods

Study design and ethical considerations

This retrospective multicenter cohort pilot study was conducted using de-identified clinical records and panoramic radiographs from patients rehabilitated with FCDs at three private dental clinics in Bangkok, Thailand (designated Clinic A, Clinic B, and Clinic C; specific names withheld to preserve clinic confidentiality). Data were collected by a single trained investigator (R.U.) using a standardized case report form for the period 2017-2025. The study period was selected to capture a sufficient number of patients with documented PBL while ensuring adequate follow-up duration. From an initial pool of 89 assessed patients, 32 met all inclusion criteria. The study was approved by the institutional ethics committee (approval: 681109) and conducted in accordance with the Declaration of Helsinki. As the study involved retrospective analysis of existing clinical records with no direct patient contact, the ethics committee waived the requirement for individual informed consent. This report adheres to the STROBE guidelines for observational cohort studies. Formal sample size calculation was not performed a priori, as no prior study provided effect-size estimates; this study is positioned as exploratory signal detection.

Data extraction used a standardized case report form across all centers. Radiographic PBL assessment was performed by a single calibrated examiner (R.U.); intraexaminer reliability was formally quantified. Descriptive statistics, univariate logistic regression, Fisher's exact and Fisher-Freeman-Halton tests, Mann-Whitney U tests, and Kaplan-Meier survival analysis were performed using IBM SPSS Statistics for Windows, Version 27 (Released 2019; IBM Corp., Armonk, New York, United States). Firth's penalized logistic regression, including Wald-type 95% confidence intervals, the penalized likelihood-ratio test, and bootstrap resampling (2,000 replications), was performed in R version 4.5.1 (R Foundation for Statistical Computing, Vienna, Austria) using the logistf package version 1.26.1 (Heinze and Ploner). Two-sided P < .05 was considered statistically significant.

Study population and eligibility criteria

Patients treated with FCDs supported by four or six implants per arch between 2017 and 2025 were screened across three centers.

Inclusion Criteria

The inclusion and exclusion criteria are listed in Table 1. Patients were included if they met all of the following criteria: (1) rehabilitated with All-on-4 or All-on-6 FCDs; (2) complete clinical records available, including demographics, medical history, implant specifications, and documentation of all prosthetic maintenance events; (3) panoramic radiographs available from implant placement visits; and (4) a minimum total follow-up period of at least 12 months from the date of implant placement (selected to allow sufficient time for initial osseointegration and a period of functional prosthetic loading before outcome assessment, consistent with reporting conventions in the FCD complication literature [3-8]; this criterion applies to total observation duration rather than time to event).

**Table 1:** Inclusion and exclusion criteria for case selection FCDs: fixed complete dentures; PBL: periapical bone loss. The minimum 12-month follow-up was selected to allow sufficient time for initial osseointegration and prosthetic loading before outcome assessment. Patients meeting all inclusion criteria but presenting any exclusion criterion were not enrolled. The exclusion of patients without periapical bone loss reflects the case-only design of this pilot study; future confirmatory studies should include PBL-free arches as comparators.

Inclusion Criteria	Exclusion Criteria
Rehabilitated with All-on-4 or All-on-6 FCDs (four or six implants per arch)	Patients aged <18 years at implant placement
Complete clinical records available, including demographics, medical history, implant specifications, and documentation of all prosthetic maintenance events	Uncontrolled diabetes mellitus, osteoporosis under intravenous bisphosphonate therapy, or a history of head and neck radiotherapy or chemotherapy
Panoramic radiographs available from implant placement and follow-up visits	Absence of periapical bone loss on follow-up panoramic radiographs (case-only design)
Minimum total follow-up of ≥12 months from the date of implant placement	

Exclusion Criteria

Patients were excluded if they met any of the following criteria: (1) age below 18 years at the time of implant placement; (2) uncontrolled diabetes mellitus, osteoporosis under intravenous bisphosphonate therapy, or a history of head and neck radiotherapy or chemotherapy; and (3) absence of periapical bone loss on follow-up panoramic radiographs, reflecting the case-only design of this pilot study.

**Figure 1:**
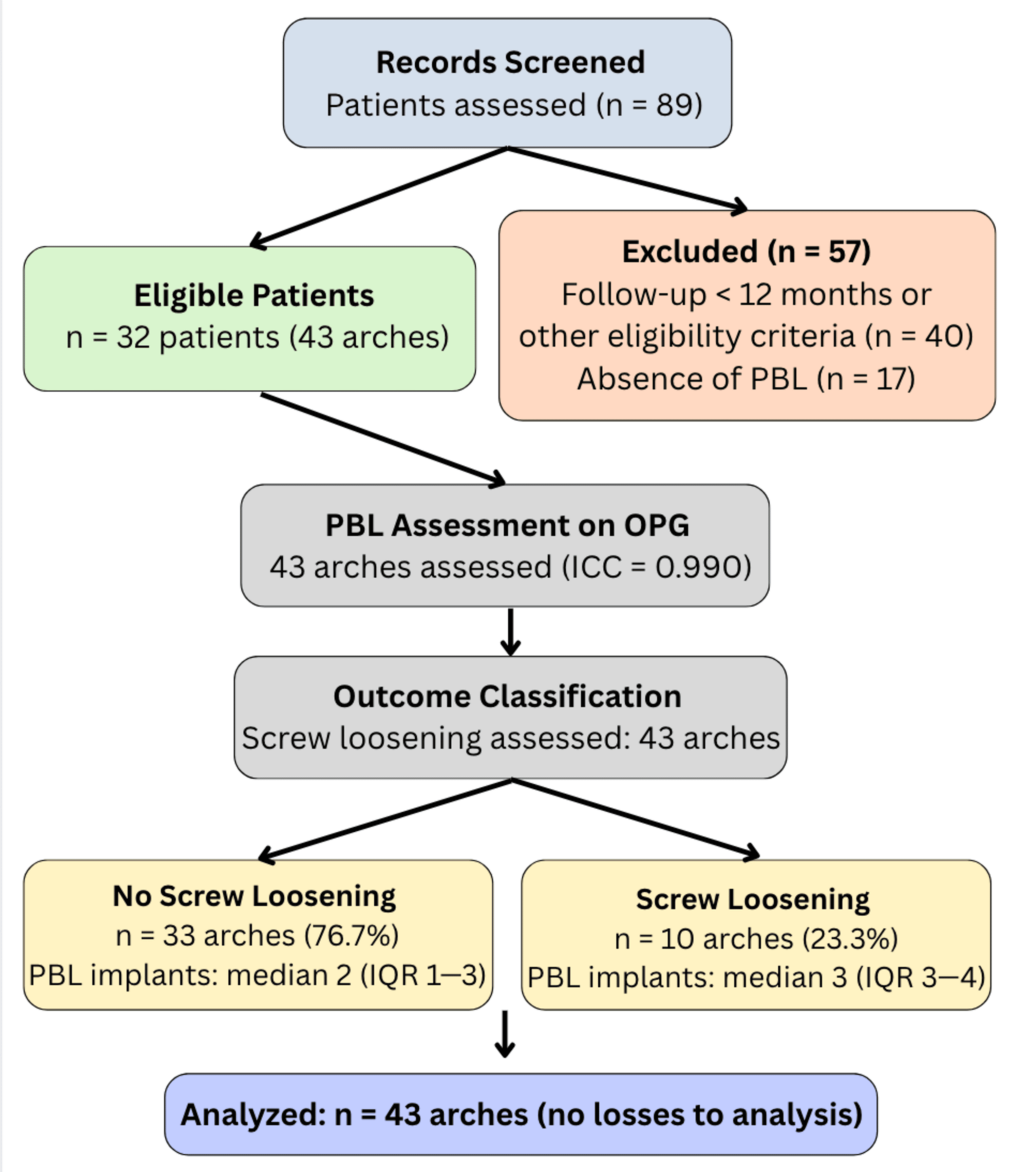
STROBE-compliant participant flow diagram STROBE-compliant flow diagram of participant selection and outcome classification. From 89 patients initially assessed for eligibility, 57 were excluded (follow-up <12 months or did not meet other eligibility criteria, n=40; absence of periapical bone loss on follow-up panoramic radiographs, n=17), yielding 32 eligible patients contributing 43 dental arches. All 43 arches were assessed for periapical bone loss on panoramic radiographs by a single calibrated examiner (intraclass correlation coefficient (ICC)=0.990) and subsequently classified by prosthetic screw loosening status. Median (range) number of implants with PBL is shown for each outcome group. All 43 arches were retained in the analysis with no losses to follow-up. Image credit: Figure created by the authors using Microsoft PowerPoint (Microsoft Corp., Redmond, WA, USA); no generative AI image tools were used.

Of 89 initially assessed patients, 57 were excluded (follow-up <12 months, n=33; incomplete records, n=4; systemic conditions, n=3; absence of PBL, n=17), yielding 32 patients contributing 43 dental arches (Figure 1).

Radiographic assessment and definition of PBL

**Figure 2:**
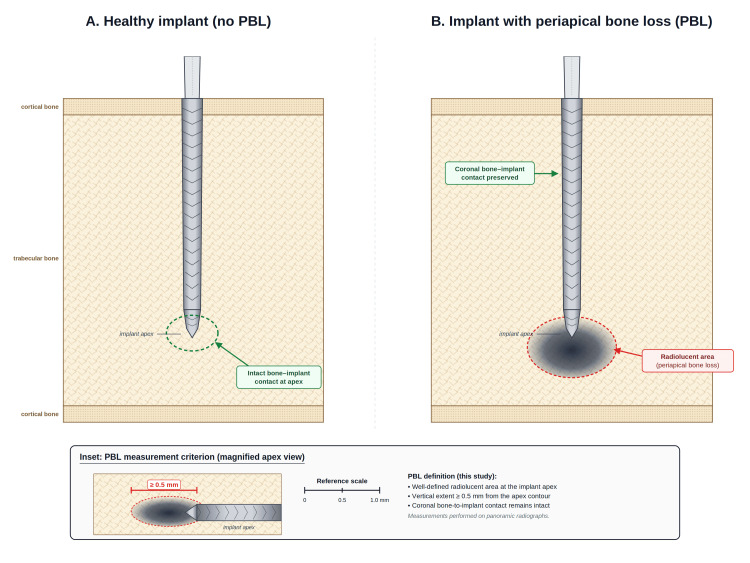
Schematic illustration of periapical bone loss measurement criterion (A) Healthy implant showing intact bone-to-implant contact at the apex. (B) Implant with periapical bone loss (PBL), showing a well-defined radiolucent area at the apex while coronal bone-to-implant contact remains preserved. The inset (lower panel) shows the magnified apex view with the measurement criterion used in this study: vertical extent ≥ 0.5 mm from the apex contour, measured on panoramic radiographs. The 0.5-mm vertical threshold was adopted as a pragmatic minimum detectable apical radiolucency on panoramic radiographs that exceeds expected measurement error operational threshold. Image credit: Figure created by the authors as a scalable vector graphic (SVG) using deterministic code rendered with CairoSVG (Python library); no generative AI image tools were used in producing this figure.

Panoramic radiography was used as the sole imaging modality because it remains the most widely available and cost-effective tool for routine implant follow-up; CBCT is frequently inaccessible in resource-constrained settings owing to equipment cost [27]. Nevertheless, orthopantomography limitations including geometric distortion and reduced sensitivity compared with CBCT may have introduced measurement imprecision, and future studies should incorporate CBCT to validate these findings. Representative measurement criteria are illustrated in Figure 2.

Definition of prosthetic screw loosening

Prosthetic screw loosening was defined as documented prosthesis mobility or screw retightening in the clinical chart, excluding fracture or implant failure. Torque verification was not systematically recorded. Loosening was recorded as binary (present/absent) at the arch level.

Predictor variables

Eleven predictor variables were recorded at baseline: (1) patient age group (≤60 vs >60 years); (2) sex; (3) presence of systemic comorbidities; (4) smoking status; (5) bruxism (documented nightguard use); (6) implant brand (three categories: NeoBiotech, Dio, and Straumann); (7) implant diameter, categorized as none, one, or two wide implants (≥5 mm, as classified by Al-Johany et al. [28]) per arch; (8) prosthesis configuration (All-on-4 vs All-on-6); (9) opposing arch type (natural dentition vs prosthesis); (10) jaw (maxilla vs mandible); and (11) cumulative PBL burden, quantified as the number of implants with PBL per arch and the anatomical position of PBL-affected implants (anterior only, posterior only, or both regions).

Statistical analysis

Descriptive statistics characterized patient- and arch-level variables. Categorical variables were compared using Fisher's exact test (for two-category variables) or the Fisher-Freeman-Halton exact test (for three-category variables); the number of PBL-affected implants and follow-up duration were compared using the Mann-Whitney U test, as non-normality was confirmed by the Shapiro-Wilk test. Univariate binary logistic regression was performed for each predictor.

Multivariable analysis was conducted using Firth's penalized logistic regression as the primary model. This approach was chosen a priori, equivalent reasoning given the low events-per-variable ratio (EPV=5.0) and the anticipated risk of quasi-separation in small samples with categorical predictors. Conventional maximum-likelihood logistic regression was attempted but failed to converge owing to quasi-separation, supporting the appropriateness of the penalized approach. Variables with univariate P<.20 were considered as candidates; however, the multivariable model was restricted to PBL-related variables (number of PBL-affected implants and PBL position) to preserve analytical parsimony given the low EPV. ORs are reported with Wald-type 95% CIs, and P-values were derived from the penalized likelihood-ratio test. Discriminative performance was evaluated by the area under the receiver operating characteristic curve (AUC). Bootstrap resampling with 2,000 replications was used as an additional sensitivity analysis. Kaplan-Meier survival analysis with the log-rank (Mantel-Cox) test was performed as a supplementary time-to-event analysis.

## Results

Study population and baseline characteristics

Thirty-two patients (43 arches) were included. Of the 32 patients, 21 contributed a single arch, and 11 contributed two arches each (paired maxillary and mandibular), yielding 43 arches. Screw loosening occurred in 10 arches (23.3%). The intraexaminer intraclass correlation coefficient (ICC) was 0.990 (95% CI:0.978-0.996). Baseline characteristics are summarized in Table 2. No significant differences were observed between groups for demographic or clinical variables (all P>.10), except implant diameter (P=.047) and number of PBL-affected implants (median 3 vs 2; P=.005).

**Table 2:** Baseline demographic and clinical characteristics stratified by prosthetic screw loosening status Data are reported at the arch level (N = 43 arches from 32 patients). Loosening group, n = 10 arches (8 patients); No Loosening group, n = 33 arches (24 patients). Data are presented as n (%) for categorical variables and median (IQR; P25–P75) for continuous variables. Statistical tests: * Fisher's exact test (two-category variables); † Fisher–Freeman–Halton exact test (≥three-category variables); ‡ Mann–Whitney U exact test (continuous variables; non-normality confirmed by the Shapiro–Wilk test, P < .05 in ≥1 group). § Age is a patient-level characteristic (N = 32 patients); data were available for 31 of 32 patients (one missing record), and the median (IQR) was based on n = 31. P-values in bold indicate statistical significance (P < .05). Eleven of 32 patients contributed two (bilateral) arches; the remaining 21 contributed one arch each. IQR: interquartile range; PBL: periapical bone loss.

Characteristic	Total (N = 43)	Loosening	No Loosening	P-value
Demographics				
Age, years, median (IQR) §	52 (45–62)	53 (45–62)	52 (43–62)	.949 ‡
Sex				
Male	20 (46.5%)	4 (40.0%)	16 (48.5%)	.727 *
Female	23 (53.5%)	6 (60.0%)	17 (51.5%)	
Systemic conditions				
Diabetes mellitus	4 (9.3%)	1 (10.0%)	3 (9.1%)	1.000 *
Osteoporosis	3 (7.0%)	0 (0.0%)	3 (9.1%)	.559 *
Bruxism	9 (20.9%)	3 (30.0%)	6 (18.2%)	.408 *
Smoking	7 (16.3%)	1 (10.0%)	6 (18.2%)	.677 *
Implant and prosthesis characteristics				
Implant brand				
NeoBiotech	21 (48.8%)	4 (40.0%)	17 (51.5%)	.531 †
Dio	13 (30.2%)	4 (40.0%)	9 (27.3%)	
Straumann	9 (20.9%)	2 (20.0%)	7 (21.2%)	
Implant diameter group				
No wide-diameter implant (≥5 mm)	17 (39.5%)	4 (40.0%)	13 (39.4%)	.984 †
1 wide-diameter implant	16 (37.2%)	4 (40.0%)	12 (36.4%)	
≥2 wide-diameter implants	10 (23.3%)	2 (20.0%)	8 (24.2%)	
Number of implants per arch				
Four implants (All-on-4)	21 (48.8%)	5 (50.0%)	16 (48.5%)	.925 *
Six implants (All-on-6)	22 (51.2%)	5 (50.0%)	17 (51.5%)	
Opposing dentition				
Natural teeth	27 (62.8%)	5 (50.0%)	22 (66.7%)	.471 *
Denture/prosthesis	16 (37.2%)	5 (50.0%)	11 (33.3%)	
Arch				
Maxillary	19 (44.2%)	4 (40.0%)	15 (45.5%)	1.000 *
Mandibular	24 (55.8%)	6 (60.0%)	18 (54.5%)	
Periapical bone loss (PBL)				
No. of implants with PBL per arch, median (IQR)	2 (1–3)	3 (3–4)	2 (1–3)	.005 ‡
PBL location				
Anterior only	16 (37.2%)	2 (20.0%)	14 (42.4%)	.258 †
Posterior only	10 (23.3%)	3 (30.0%)	7 (21.2%)	
Both (anterior + posterior)	17 (39.5%)	5 (50.0%)	12 (36.4%)	
Follow-up				
Total follow-up duration, months, median (IQR)	16.7 (14.4–23.9)	15.1 (12.2–18.2)	17.1 (14.4–24.9)	.089 ‡

Univariate logistic regression analyses

Univariate logistic regression results are shown in Table 3. The number of PBL-affected implants was the only significant continuous predictor (OR=2.539, 95% CI:1.200-5.371, P=.015). The implant diameter reached omnibus significance (P=.026) with a dose-dependent trend. Although the PBL position reached the univariate omnibus screening threshold (P=.033, Table 3), this is an unadjusted association; after adjustment for the number of PBL-affected implants, it was not independently significant (Table 4, P=.475), indicating that the univariate signal is attributable to its correlation with PBL burden.

**Table 3:** Univariate logistic regression analyses for predictors of prosthetic screw loosening (n=43 dental arches) *P < .05 (statistically significant). †.05 ≤ P < .20 (met screening threshold for multivariable candidacy; only PBL-related variables were retained owing to the low EPV). The omnibus P shown for three-category variables is a univariate screening value, not an inferential test of independent significance. For three-category variables (diameter, PBL position, brand), the omnibus likelihood-ratio χ² and overall model P are shown; B, Wald, and OR are shown only for two-category variables. OR: odds ratio; CI: confidence interval; B: logistic regression coefficient; EPV: events-per-variable ratio; PBL: periapical bone loss.

Predictor Variable	Omnibus χ²	df	p (model)	Nagelkerke R²	B	Wald	p (coeff.)	OR (95% CI)
No. of implants with PBL*	7.244	1	.007*	.234	0.932	5.942	.015*	2.539 (1.200-5.371)
Implant diameter (3-cat)*	4.978	2	.026*	.165	—	—	—	—
PBL position (3-cat)†	4.555	2	.033	.152	—	—	—	—
Jaw (maxilla vs mandible)†	3.302	1	.069	.112	−1.447	2.805	.094	0.235 (0.043-1.279)
Smoking	0.714	1	.398	.025	1.269	0.751	.386	3.556 (0.202-62.632)
All-on-4 vs All-on-6	0.410	1	.522	.014	0.466	0.404	.525	1.594 (0.379-6.711)
Bruxism	0.035	1	.852	.001	−0.216	0.034	.855	0.806 (0.080-8.160)
Opposing arch	0.405	1	.525	.015	0.553	0.398	.528	1.739 (0.312-9.694)
Sex	0.650	1	.420	.023	−0.588	0.641	.488	0.556 (0.132-2.342)
Systemic disease	0.039	1	.844	.001	−0.154	0.039	.844	0.857 (0.185-3.974)
Age group	0.142	1	.706	.005	−0.288	0.142	.717	0.750 (0.168-3.351)
Brand (3-cat)	0.500	2	.779	.018	—	—	—	—

**Table 4:** Firth's penalized multivariable logistic regression analysis for predictors of prosthetic screw loosening Firth's penalized maximum-likelihood estimation (n = 43 dental arches). Firth's penalized regression was used as the primary multivariable analysis because conventional maximum-likelihood logistic regression failed to converge owing to quasi-separation (zero loosening events in the "anterior only" PBL position category, n = 7), and because the low events-per-variable ratio (EPV = 5.0) increased the risk of small-sample bias in standard logistic regression. Firth's penalization (Jeffreys-prior bias correction) provides asymptotically unbiased and finite estimates under these conditions. P-values are derived from the penalized likelihood-ratio test; the 95% CIs shown are Wald-type intervals (B ± 1.96 × SE). In small, quasi-separated samples, the penalized likelihood-ratio test provides more reliable inference than the Wald interval, which may marginally include unity even when the corresponding test is significant. Wald-type intervals (B ± 1.96 × SE) assume a symmetric normal approximation that performs poorly under sparse events and near-separation, where the likelihood is asymmetric; the penalized likelihood-ratio test evaluates the actual change in penalized log-likelihood and is therefore preferred here. The bootstrap CI (1.117–7.229), which excludes unity, is consistent with the likelihood-ratio result. Model performance: penalized likelihood-ratio test χ² = 8.71, df = 2, P = .013; area under the ROC curve (AUC) = 0.776 (95% CI: 0.632–0.920, P = .009); overall classification accuracy = 74.4% at a probability cut-off of 0.5. *P < .05 (statistically significant, by penalized likelihood-ratio test). OR: odds ratio; CI: confidence interval; B: logistic regression coefficient; SE: standard error. —: not applicable; PBL: periapical bone loss.

Predictor variable	B	SE	OR	95% CI Lower	95% CI Upper	P-value	Note
No. of implants with PBL	0.748	0.483	2.113	0.820	5.439	.037*	Primary finding
PBL position (ordinal)	0.156	0.800	1.169	0.244	5.610	.475	
Constant	-3.381	1.628	0.034	—	—	.038*	

Multivariable logistic regression analysis

Firth's penalized logistic regression was selected as the primary multivariable analysis instead of conventional maximum-likelihood logistic regression. Two factors motivated this choice. First, the conventional model failed to converge owing to quasi-separation: the "anterior only" PBL location category (n=7) contained zero loosening events, producing extreme coefficient estimates with implausibly large standard errors. Second, the low EPV (5.0, below the recommended minimum of 10 [29]) increases the risk of small-sample bias in standard logistic regression. Firth's penalization, which uses Jeffreys-prior bias correction, provides asymptotically unbiased and finite estimates under both conditions and is the recommended approach for rare-event and separated data.

Among the non-PBL predictors, only jaw type met the P < .20 univariate screening threshold; it was nonetheless excluded from the primary multivariable model owing to the low EPB (≈ 5.0, below the recommended minimum of 10) and to preserve analytical parsimony focused on the primary research question. The exclusion of this potential confounder is a recognized limitation; future adequately powered studies should incorporate it, together with other recognized confounders such as smoking and bruxism, in fully adjusted models.

**Figure 3:**
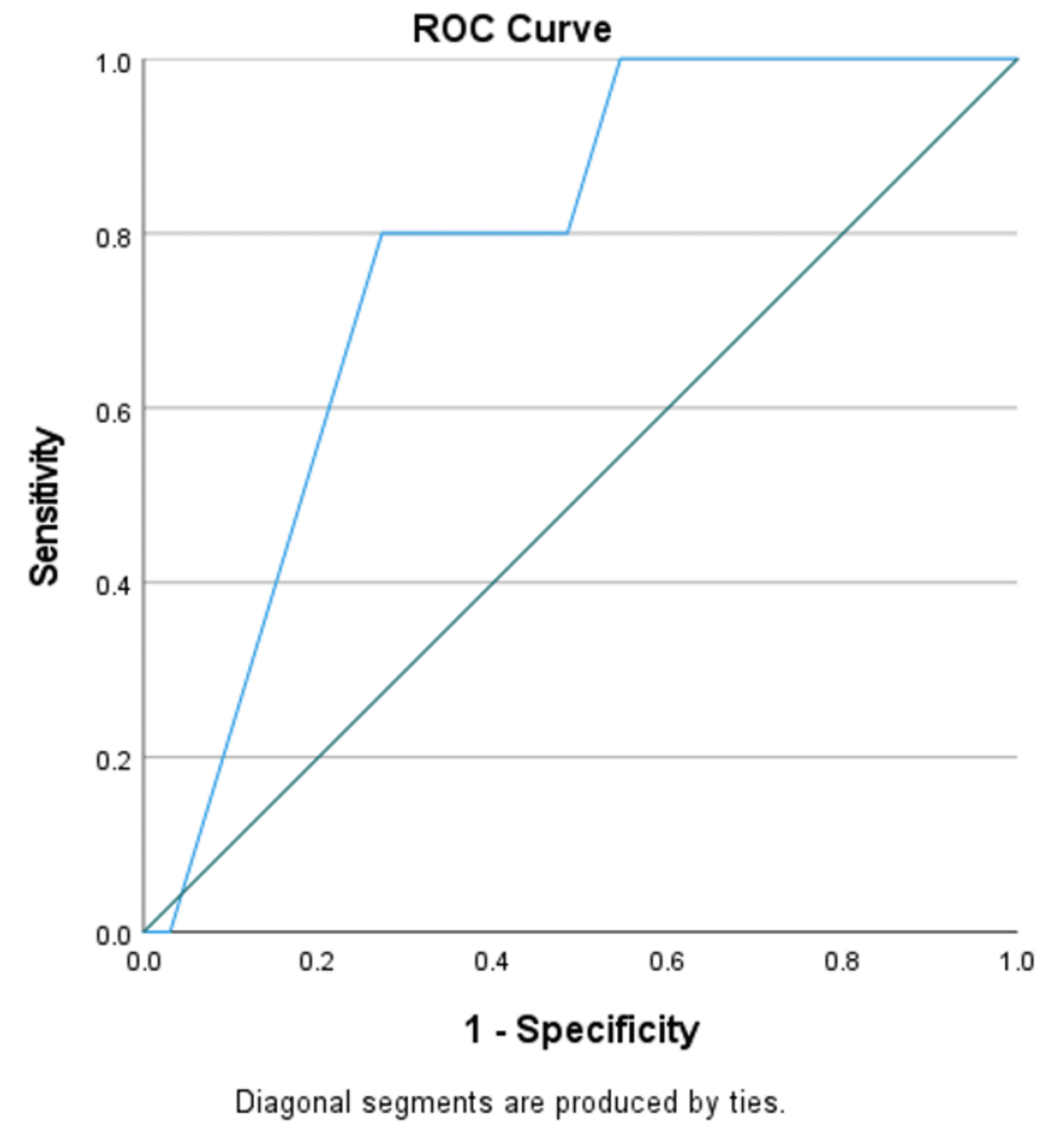
Receiver operating characteristic (ROC) curve for the multivariable model predicting prosthetic screw loosening ROC curve illustrating the discriminative performance of the multivariable logistic regression model (predictors: number of implants with periapical bone loss and PBL position) for predicting prosthetic screw loosening among 43 dental arches (10 loosening events). The blue curve represents the model; the diagonal reference line represents chance discrimination (area under the curve (AUC)=0.500). AUC = 0.776 (95% CI: 0.632–0.920; P=.009), indicating acceptable discrimination. Predicted probabilities were derived from standard maximum-likelihood logistic regression and used for ROC analysis; the rank-order of predicted probabilities is invariant to the choice between standard and Firth's penalized estimation, so the AUC value reflects discrimination of the multivariable predictor set as a whole. Diagonal segments in the curve indicate ties in predicted probabilities. The ROC analysis was performed on the full analytic sample of 43 dental arches (10 loosening events) and was not stratified into PBL-burden groups; ROC analysis performed using IBM SPSS Statistics version 27.0. PBL: Periapical bone loss

The penalized multivariable model was statistically significant (penalized likelihood ratio test χ²=8.71, df=2, P=.013), and discriminative performance was acceptable, with an AUC of 0.776 (95% CI: 0.632-0.920, P=.009; Figure 3); overall classification accuracy was 74.4% at a probability cut-off of 0.5. Multivariable results are presented in Table 4.

After adjustment for PBL position, the number of PBL-affected implants showed a positive association with prosthetic screw loosening (Firth-penalized OR = 2.113; P = .037 by penalized likelihood-ratio test), corresponding to approximately a 2.1-fold increase in the odds of screw loosening for each additional PBL-affected implant per arch. The corresponding Wald-type 95% CI (0.820-5.439) marginally included unity; this discordance between the Wald interval and the penalized likelihood-ratio test is expected in small, quasi-separated samples, in which the penalized likelihood-ratio test provides more reliable inference than the Wald interval. PBL position was not associated with screw loosening (OR = 1.169; 95% CI: 0.244-5.610; P = .475).

To place the magnitude of the association in clinical context, the absolute event rate was 47.1% (8 of 17 arches) in Group B (≥3 PBL-affected implants) versus 7.7% (2 of 26 arches) in Group A (1-2 PBL-affected implants), representing an absolute risk difference of 39.4 percentage points (95% CI: 13.6-65.2), corresponding to a number needed to harm (NNH) of 2.5 (95% CI: 1.5-7.4). This NNH was a post hoc calculation, not a prespecified analysis, computed to contextualize the dose-response signal; given the small sample and wide interval, it should be read as an illustrative effect-size descriptor rather than a precise clinical estimate. This means that for every 2.5 arches with high PBL burden (≥3 affected implants) versus low burden (1-2 affected implants), one additional screw loosening event would be expected. The NNH should be interpreted with caution, given the small sample size (n=43 arches, 10 events), which produces wide confidence intervals and limits the precision of absolute risk estimates.

Kaplan-Meier survival analysis

Kaplan-Meier survival analysis was performed as a supplementary time-to-event analysis (Table 5). Screw loosening events occurring before 12 months were observed in 3 of 10 affected arches and reflect early mechanical failure within adequate overall follow-up, not a violation of the inclusion criterion, as the minimum criterion applies to total observation duration rather than time to event.

**Table 5:** Kaplan-Meier survival analysis: time to first screw loosening stratified by the PBL burden group N: number of dental arches in each group; Events: number of arches that experienced prosthetic screw loosening during follow-up; Censored: number of arches without screw loosening at the end of follow-up (right-censored observations). Mean survival reported; median could not be estimated owing to the high censoring rate. Group A: 1–2 implants with PBL; Group B: ≥3 implants with PBL. CI: confidence interval; SE: standard error. —: not applicable; PBL: periapical bone loss.

PBL Group	N	Events, n (%)	Censored, n (%)	Mean survival (months)	SE	95% CI	Log-rank p
Group A (1–2 implants with PBL)	26	2 (7.7%)	24 (92.3%)	30.0	1.25	27.6–32.5	.002
Group B (≥ 3 implants with PBL)	17	8 (47.1%)	9 (52.9%)	19.5	3.31	13.0–26.0	—

**Figure 4:**
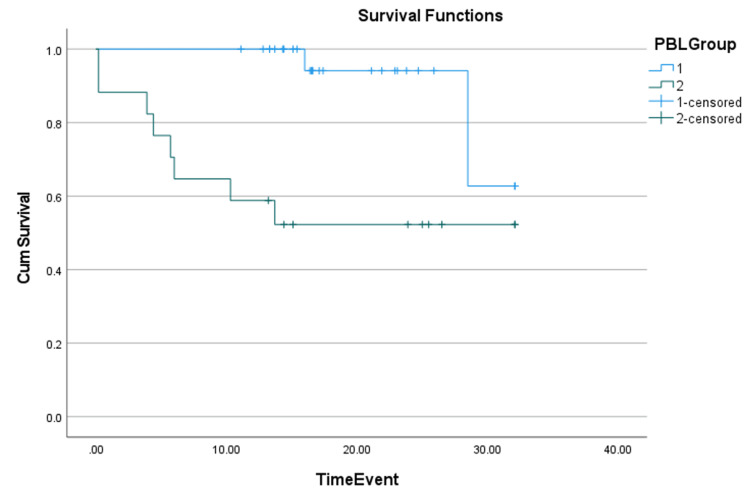
Kaplan–Meier survival curves for time to prosthetic screw loosening, stratified by periapical bone loss burden Kaplan–Meier survival curves for time to prosthetic screw loosening among 43 dental arches, stratified by periapical bone loss (PBL) burden. Group A (light blue line): 1–2 implants with PBL per arch (n=26 arches; two loosening events). Group B (dark blue line): ≥3 implants with PBL per arch (n=17 arches; eight loosening events). Tick marks indicate right-censored observations (no loosening event at the end of follow-up). The difference between groups was statistically significant (log-rank [Mantel–Cox] test: χ²=9.548, df=1, P=.002). The x-axis "TimeEvent" denotes time from prosthesis loading to first prosthetic screw loosening (or censoring), in months. Figure created by the authors using IBM SPSS Statistics for Windows, version 27.0 (IBM Corp., Armonk, NY, USA). PBL: Periapical bone loss

Group A (1-2 PBL-affected implants; n=26) had two loosening events (7.7%) with a mean loosening-free time of 30.0 months. Group B (≥3; n=17) had eight events (47.1%) with a mean of 19.5 months. The log-rank test confirmed significant separation (χ²=9.548, df=1, P=.002). The survival curves are shown in Figure 4.

Sensitivity analyses

Bootstrap resampling (2,000 replications) confirmed the univariate association between the number of PBL-affected implants and screw loosening (mean OR=2.842; 95% CI: 1.117-7.229; P=.028), with the lower bound of the 95% CI excluding the null. The convergence between the primary penalized regression and bootstrap estimates strengthens the inferential reliability of the observed association despite the small sample size.

## Discussion

Although screw loosening is among the most prevalent mechanical complications of FCDs, no study has previously examined whether periapical bone loss contributes to this outcome. The present study found that cumulative PBL burden was consistently associated with prosthetic screw loosening across multiple analytical approaches (OR=2.539, P=.015), representing a novel but preliminary observation.

The observed 23.3% loosening rate falls within the 2.8% to 33% range reported in the FCD literature, depending on follow-up duration, loading protocol, and prosthetic design [3-8]. However, because this study employed a case-only design excluding PBL-free arches, the elevated rate cannot be directly attributed to PBL; it may partly reflect cohort characteristics, including referral patterns at the participating clinics. Establishing whether PBL independently elevates screw-loosening risk will require controlled comparison with PBL-free arches in future studies.

Implant diameter also differed significantly between groups (P=.047). When PBL coexists at wide-implant sites, reduced anchorage may compound bending moments at the screw interface [22], though this association requires further investigation given the low EPV ratio.

The dose-response pattern supports a hypothesis that cumulative PBL has compounding biomechanical consequences: apical bone loss reduces anchorage length, amplifies bending moments, and accelerates preload loss [20-22]. Kaplan-Meier analysis corroborated this with a six-fold higher event rate in Group B (47.1% vs 7.7%), though small-sample survival estimates should be interpreted cautiously. However, existing finite-element evidence supporting this pathway derives predominantly from models simulating progressive marginal bone loss [22]. Apical defects affect a region geometrically distant from the screw joint and may alter stress distribution in ways not captured by current models. No finite-element analysis evaluating the mechanical consequences of periapical lesions on screw-joint integrity has been published, and thus, the proposed mechanism remains inferential. To date, the PBL-to-preload pathway has not been demonstrated clinically; it remains a plausible but untested hypothesis, and the present association represents the first clinical signal rather than confirmation of the mechanism.

In the penalized model, the number of PBL-affected implants showed a positive association with screw loosening (OR = 2.113; P = .037 by the penalized likelihood-ratio test) after adjustment for PBL position. The accompanying Wald-type 95% CI (0.820-5.439) marginally included unity, consistent with the limited statistical power expected at an events-per-variable ratio of 5.0 [29]; bootstrap resampling (2,000 replications) yielded a higher mean OR (2.842; 95% CI: 1.117-7.229; P = .028) with a 95% CI excluding the null, providing further support for the observed association. With only 10 loosening events, even the penalized model has limited stability, and point estimates should be regarded as preliminary effect-size signals for sample-size planning rather than reliable risk estimates.

The PBL position was nonsignificant across analyses, possibly because cross-arch splinting redistributes loads across the full-arch framework [23]. However, finite-element analysis suggests that implant position and framework rigidity modulate load distribution [22], warranting further investigation. Similarly, null findings for other predictors likely reflect insufficient power (10 events in 43 arches) [4,29] rather than true absence of association; low documented smoking and bruxism prevalence may reflect chart-review underreporting.

Although no prior study has directly linked PBL to screw loosening, adjacent evidence supports the proposed pathway. Studies on marginal bone loss have demonstrated that progressive crestal resorption concentrates stress at the implant-abutment junction, accelerating preload degradation [22]. Conversely, screw loosening enlarges the implant-abutment microgap, facilitating bacterial colonization that promotes further bone resorption [24,25]. These converging lines of evidence are consistent with the bidirectional relationship proposed earlier, whereby PBL and screw loosening may form a self-reinforcing cycle. However, the cross-sectional nature of this study precludes determination of temporal sequence, and the bidirectional hypothesis remains to be tested through longitudinal investigation. Specifically, this design cannot establish whether PBL preceded screw loosening or the reverse: screw loosening may itself contribute to bone loss by enlarging the implant-abutment microgap and promoting micromotion and microbial colonization [24,25]. Resolving temporality requires a longitudinal study with PBL assessment anchored to the implant-placement radiograph.

If confirmed, progressive PBL may serve as an early radiographic indicator of impending mechanical failure [11,13], suggesting that clinicians should implement closer monitoring protocols, including periodic torque verification in patients presenting with multiple PBL-affected implants supporting FCDs [9,12]. In resource-constrained settings where panoramic radiographs remain the primary surveillance tool [27], identifying PBL as a risk marker could facilitate timely intervention without requiring advanced imaging.

As a retrospective observational study, this analysis can demonstrate association only and cannot confirm a cause-and-effect relationship between periapical bone loss and prosthetic screw loosening. Several limitations warrant consideration. The retrospective design introduces potential information bias inherent to chart-based data extraction. PBL was assessed on panoramic radiographs using a 0.5-mm threshold, which may underestimate true prevalence compared with CBCT owing to geometric distortion and superimposition [30]. The single-examiner design, while yielding excellent intra-examiner reliability (ICC=0.990), precludes assessment of inter-examiner agreement. Eleven patients contributed two arches each; maxillary and mandibular arches from the same patient share host-level factors, partially violating the independence assumption. With only 32 patients and 10 events, a mixed-effects or generalized estimating equation model with a patient-level random effect could not be reliably fitted. Reported estimates therefore ignore potential intracluster correlation and may understate standard errors; confirmatory studies should employ patient-level clustering adjustment. Potentially important confounders, including smoking, bruxism, and parafunctional habits, were inadequately captured; smoking in particular may have biased PBL effect estimates. Unmeasured biomechanical variables (occlusal force magnitude, insertion torque, bone density) could not be accounted for. The lack of standardized torque verification across clinics may affect the accuracy of screw-loosening assessment: loosening was identified from documented mobility or chart-recorded retightening rather than standardized torque measurement, which may cause both under-detection of subclinical preload loss and between-center variability. Because PBL-free arches were excluded by design, this study cannot estimate the risk attributable to the presence of PBL itself; it evaluates only the dose-response relationship across differing PBL burden among already-affected arches. Consequently, this study cannot directly compare screw-loosening risk between arches with and without PBL, and the 23.3% loosening rate cannot be attributed to PBL. Finally, recruitment from three private clinics in a single metropolitan area limits generalizability.

Based on the observed effect size, a confirmatory multicenter prospective cohort study enrolling 150-220 arches, including PBL-free controls, is recommended. Such a study should employ cone-beam computed tomography for three-dimensional PBL assessment, standardize torque verification protocols, and capture biomechanical confounders including occlusal force and bone density. The convergence across univariate, Firth's penalized, bootstrap, and Kaplan-Meier analyses strengthens the present signal and supports this larger confirmatory investigation.

## Conclusions

Within the limitations of this retrospective pilot study, the following conclusions were drawn. First, in this exploratory pilot, the number of periapical bone loss-affected implants per arch was associated, as a hypothesis-generating observation, with prosthetic screw loosening in full-arch FCDs. Second, arches with three or more affected implants showed shorter screw-loosening-free survival than those with fewer affected implants. Third, these exploratory findings require confirmation in adequately powered prospective studies.
